# Use of ^99m^ Tc-DTPA Scintigraphy in Evaluation of Ureteral Laceration Due to Transurethral Lithotripsy in a Patient with Nephrolithiasis

**DOI:** 10.1055/s-0042-1757282

**Published:** 2022-10-28

**Authors:** Esmaeil Gharepapagh, Ashraf Fakhari, Afshar Zomorrodi, Shahram Dabiri Oskuei

**Affiliations:** 1 Medical Radiation Sciences Research Team, Tabriz University of Medical Sciences, Tabriz, Iran; 2 Department of Urology, Tabriz University of Medical Sciences, Tabriz, Iran

**Keywords:** radiopharmaceutical scan, ^99m^ Tc-DTPA renal scintigraphy, nephrolithiasis, transurethral lithotripsy, ureteroscopy

## Abstract

Transurethral lithotripsy (TUL) procedure via ureteroscopy as an invasive method for nephrolithiasis treatment would lead to urinary tract injuries. In this reported case, the procedure caused severe damage to the left ureter that was detected by ^99m^ Tc-diethylenetriaminepentaacetic acid ( ^99m^ Tc-DTPA) scan. Generally, the TUL procedure through the ureter scope is used to manage urinary tract stones. In this case, the TUL was performed on a patient with a history of nephrolithiasis. Following that, she was accompanied with abdominal pain and discomfort, so ^99m^ Tc-DTPA scintigraphy was performed to evaluate the urinary tract system. The scintigraphy showed a severe damage to the left ureter that finally resulted in autotransplantation. The control ^99m^ Tc-DTPA scintigraphy performed 3 weeks after revealed no visible urinary leakage. In this case, the ^99m^ Tc-DTPA scan prevented the patient from dangerous complications. So, ^99m^ Tc-DTPA scan could be performed after TUL and ureteroscopy to detect probable risks.

## Introduction

Nephrolithiasis, making of calculi in the kidneys or urinary tract, is one of the common kidney disorders (problems). About 9% of women and 19% of men are involved with nephrolithiasis during their lifetime. There are two common complications associated with nephrolithiasis including UTI (urinary tract infection) and CKD (chronic kidney disease). 1 TUL (transurethral lithotripsy) is a procedure to break up stones of ureters through the urethra by a ureter scope (ureteroscopy). The TUL is classified into three types including flexible, rigid, and semirigid ureteroscopes. 2 The complications of ureteroscopy are considered as major and minor types. 3 ^99m^ Tc-diethylenetriaminepentaacetic acid ( ^99m^ Tc-DTPA) renal scintigraphy is used to assay urinary excretion and drainage in addition to the glomerular filtration rate (GFR) estimation, because 90 to 95% of ^99m^ Tc-DTPA is filtrated by the glomerulus. According to functional characteristic of ^99m^ Tc-DTPA scintigraphy, renal excretory disorders especially ureteral leakage could be detected during this procedure. 4

## Case Report

**Fig. 1:**
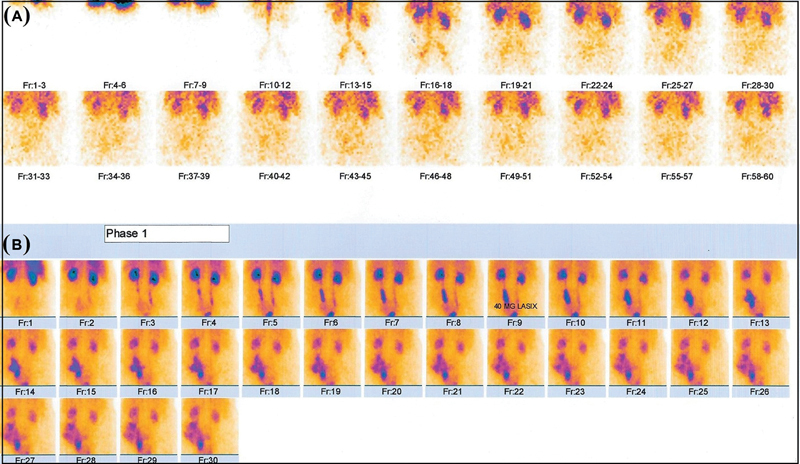
( **A** ) The perfusion phase of scintigraphy (for 60 seconds after injection of ^99m^ Tc-DTPA) shows mild decreased perfusion of both the kidneys. ( **B** ) The excretory phase reveals normal excretion of activity from the right kidney to the bladder and remarkable urinary leakage around the left ureter.

**Fig. 2:**
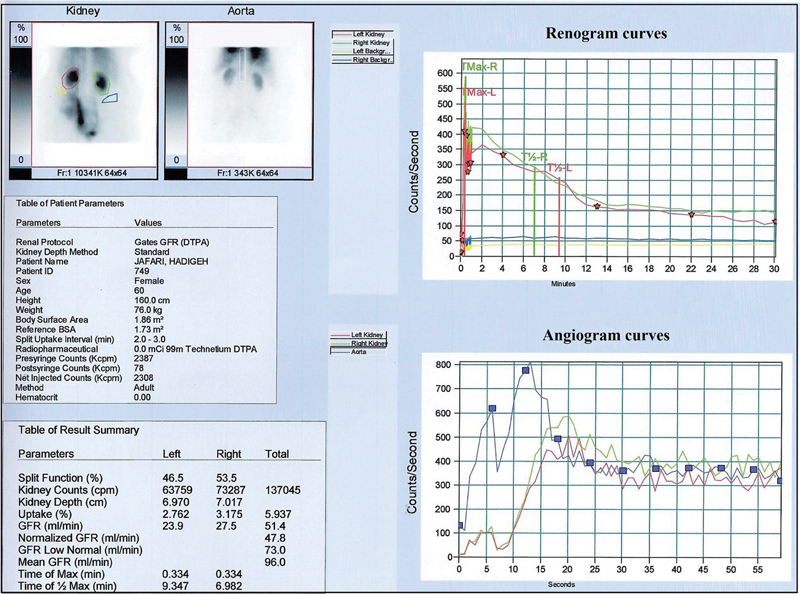
The angiogram curves illustrate mild-to-moderate decreased perfusion of both the kidneys, and the renogram curves show normal excretory function of both the kidneys despite severe ureteral damage on the left side.

**Fig. 3:**
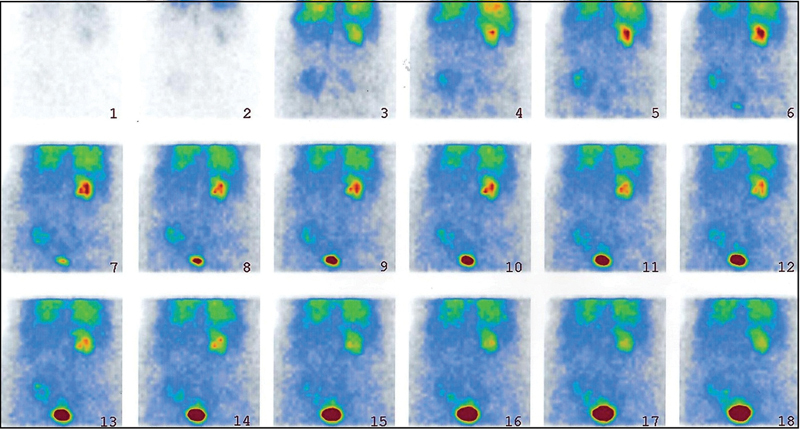
The control ^99m^ Tc-DTPA renal scintigraphy shows normal pattern of excretion in both the kidneys with no evidence of urinary leakage in the left side.

**Fig. 4:**
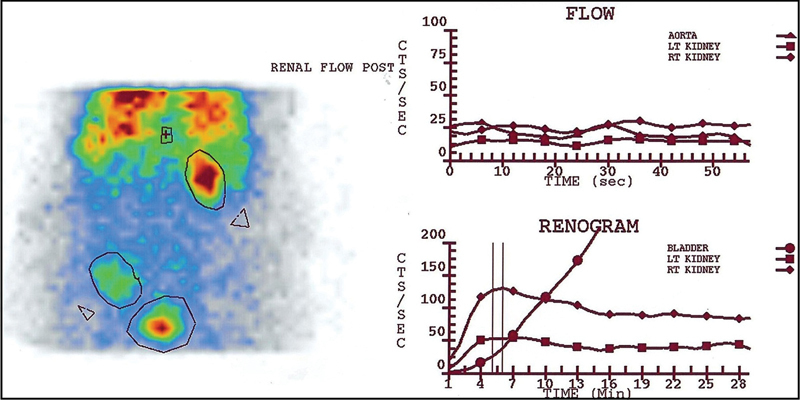
The control angiogram and renogram curves illustrate mild-to-moderate decreased perfusion and uptake and normal excretory function of both the kidneys without any evidence of urinary leakage.

In this report, a 56-year-old female patient with a history of kidney and ureteral stones who was referred to TUL procedure via ureteroscopy, presented with severe abdominal pain due to device manipulation. For this reason, the ^99m^ Tc-DTPA renal scintigraphy was performed to evaluate the urinary tract system. The renal scintigraphy by i.v. injection of 555 MBq of ^99m^ Tc-DTPA revealed mild-to-moderate decreased perfusion and function for both kidneys, especially of the left side. Moreover, an abnormal retention of radioactive urine was detected during the excretory phase of scintigraphy in the left hemiabdomen and pelvis because of left ureter urinary leakage. The urinary leakage was more considerable after i.v. injection of 0.3 mg/kg furosemide ( Fig. 1 ). According to the dynamic phase of the scan, the curve of renogram showed mild decreased perfusion and uptake of kidneys with near to normal excretory function especially after injection of a diuretic. Moreover, the renogram curves showed that there was a normal pattern of excretion on the left side despite ureteral damage, and there was mildly decreased GFR for both the kidneys ( Fig. 2 ). It should be mentioned that in spite of unsuccessful TUL, the normal urine excretion of the left kidney was related to the urine leakage into the retroperitoneal cavity that would have caused peritoneal or systemic infection. After detection of severe ureteral damage, the patient was referred to the left kidney autotransplantation surgery in the left iliac fossa and the results were evaluated by control ^99m^ Tc-DTPA renal scintigraphy 3 weeks after surgery. The control scintigraphy showed the normal drainage of urine from pelvic left kidney to the bladder without evidence of urinary leakage ( Figs. 3 and 4 ). Based on this study, it is distinguished that ^99m^ Tc-DTPA scintigraphy will be beneficial just after TUL procedure via ureteroscopy for early detection and treatment of probable complications.

## Discussion

Kidney stone is defined as the deposition of minerals in the renal parenchyma especially in calyces and pelvis. There are some types of stones including calcium oxalate, calcium phosphate, struvite, uric acid, and cysteine. 5 The TUL and SWL (shock wave lithotripsy) are modalities for the treatment of large and smaller ureteral stones, respectively. 6 According to potential risks of ureteroscopy, the urinary tract study is so important for early prognosis and treatment of complications, especially perforation and avulsion. Some diagnostic methods, for instance, ultrasound, computed tomography, magnetic resonance imaging, and renal scintigraphy could be used for kidney evaluation. 7 Among them, renal scintigraphy and functional MRI are considered as a functional imaging method. Beatrice et al showed that there are not significant differences between the two functional imaging methods in patients with drainage. 8 Renal scintigraphy, which is classified into static and dynamic imaging, has been used with different indications. 9 In this case, the TUL via ureteroscopy was used for the treatment of the patient with nephrolithiasis. Because the ureteroscopy is accompanied by some complications, the evaluation of the urinary tract could be recommended after this procedure. For this purpose, renal radiopharmaceuticals could be useful (through excretion or filtration) for the diagnosis of every ureteral damage as well as ureter perforation and avulsion. Dynamic scintigraphy by glomerular ( ^99m^ Tc-DTPA, ^99m^ Tc-GH) and tubular ( ^99m^ Tc-EC, ^99m^ Tc-MAG3) radiopharmaceuticals, has been used for kidney functional study. 10 The ^99m^ Tc-DTPA, a radiopharmaceutical filtered by glomerulus, was used for this case. Based on the passage of radioactive urine, the ureters abnormalities such as obstruction, dilatation, tortuosity, and leakage are diagnosed in the excretory phase of the study. The distribution of radioactive urine around the ureter in the retroperitoneal cavity is considered as ureteral leakage. In this case, ^99m^ Tc-DTPA scintigraphy showed the distribution of radiotracer in the mid-portion of the left ureter due to ureteroscopic manipulation. The results revealed that there is a risk of ureteral injury that leads to serious complications including perforation, laceration, and even avulsion. Following that, the patient was referred to autotransplantation surgery, and then control ^99m^ Tc-DTPA scintigraphy. The ^99m^ Tc-DTPA scintigraphy is more beneficial to early diagnosis and treatment of probable risks due to urinary tract invasive producers, so many related complications especially pelvic and systemic infections could be prevented. For this patient, the ^99m^ Tc-DTPA scan confirmed a serious injury in the left ureter that led to kidney autotransplantation. According to the results, if the ^99m^ Tc-DTPA scan was performed just after ureteroscopy, the necessary treatments could promptly be used before serious threatening signs. So, evaluation via control ^99m^ Tc-DTPA scan just after ureteroscopy is firmly recommended to prevent probable complications. Also, it is suggested to include the radionuclide study in the urological guideline as a complementary step. Because of some complications during TUL via ureteroscopy, it was concluded that performing ^99m^ Tc-DTPA renal scintigraphy is useful for the detection of probable damage. Also, based on glomerular filtration of ^99m^ Tc-DTPA, there is a chance to evaluate perfusion-function and GFR for manipulated kidneys.
